# An intestinal microRNA modulates the homeostatic adaptation to chronic oxidative stress in *C. elegans*

**DOI:** 10.18632/aging.101029

**Published:** 2016-09-12

**Authors:** Masaomi Kato, Mohammed Abul Kashem, Chao Cheng

**Affiliations:** ^1^ The Laboratory of Ageing, Centenary Institute, Camperdown, NSW 2050, Australia; ^2^ Sydney Medical School, The University of Sydney, Camperdown, NSW 2050, Australia; ^3^ Department of Biomedical Data Science, Geisel School of Medicine, Dartmouth College, Lebanon, NH 03756, USA; ^4^ Department of Molecular and Systems Biology, Geisel School of Medicine, Dartmouth College, Hanover, NH 03755, USA

**Keywords:** aging, microRNA, adaptive response, oxidative stress, *C. elegans*

## Abstract

Adaptation to an environmental or metabolic perturbation is a feature of the evolutionary process. Recent insights into microRNA function suggest that microRNAs serve as key players in a robust adaptive response against stress in animals through their capacity to fine-tune gene expression. However, it remains largely unclear how a microRNA-modulated downstream mechanism contributes to the process of homeostatic adaptation. Here we show that loss of an intestinally expressed microRNA gene, *mir-60*, in the nematode *C. elegans* promotes an adaptive response to chronic – a mild and long-term – oxidative stress exposure. The pathway involved appears to be unique since the canonical stress-responsive factors, such as DAF-16/FOXO, are dispensable for *mir-60* loss to enhance oxidative stress resistance. Gene expression profiles revealed that genes encoding lysosomal proteases and those involved in xenobiotic metabolism and pathogen defense responses are up-regulated by the loss of *mir-60*. Detailed genetic studies and computational microRNA target prediction suggest that endocytosis components and a bZip transcription factor gene *zip-10*, which functions in innate immune response, are directly modulated by miR-60 in the intestine. Our findings suggest that the *mir-60* loss facilitates adaptive response against chronic oxidative stress by ensuring the maintenance of cellular homeostasis.

## INTRODUCTION

Animals have the ability to resist and adapt appropriately to internal and external perturbations, such as stress caused by metabolic or environmental changes, ensuring organismal homeostasis throughout their lifetime [1, 2]. A better understanding of the genetic basis for homeostatic adaptation is an important step to gain insight into the biology of aging; however, while we understand much about molecular mechanisms that govern transient stress response, such as stress-dependent FOXO activation [3, 4], little is known about a genetic mechanism for long-term adaptive response to stress.

MicroRNAs (miRNAs), a class of small non-protein-coding RNA species, constitute an important mechanism for gene regulation. In general, miRNAs post-transcriptionally repress the expression of target genes by directly binding to the 3′ untranslated region (3' UTR) of their messenger RNAs (mRNAs) [5, 6]. Since their first discovery in the nematode *Caenorhabditis elegans* (*C. elegans*) as developmental timing genes [7, 8], numerous studies have revealed that miRNAs are involved in nearly all biological events, including metabolic control, immune defense and disease [9-11]. We and others have observed that miRNAs are also crucial factors in lifespan determination [12-18]. Recent development of high-throughput sequencing and computational approaches has further accelerated the discovery of many miRNAs and their contributions to gene regulatory networks [12, 16, 19-23]. Despite the importance of miRNAs in gene regulation, it has been shown that genetic deletions of individual miRNAs often result in no obvious phenotype. In *C. elegans*, for example, animals lacking individual miRNAs or even all members of a miRNA family do not display grossly abnormal phenotypes under standard laboratory conditions [24, 25]. This seems to be true for some miRNAs in mouse models as well [26]. One possible explanation for these observations is that an effect caused by a miRNA deletion is masked by genetic and functional redundancies between miRNAs and their target genes. The key properties of miRNA-mediated gene regulation, such as one miRNA targeting multiple targets and one target being regulated by multiple miRNAs, and also feedback regulation, have led to the suggestion that miRNAs act to reduce fluctuations in gene expression [26-30]. Such a buffering ability of miRNAs may contribute to homeostatic adaptation in the face of environmental or metabolic perturbations during aging, although it remains poorly understood how downstream machineries modulated by miRNAs achieve this outcome.

Here we report that loss of an intestinal miRNA gene, *mir-60*, in the nematode *C. elegans* promotes an adaptive response against oxidative stress; we found that *C. elegans* animals genetically lacking *mir-60* have a dramatically extended lifespan under a mild and long-term oxidative stress condition, while their survival is not increased under a strong and transient oxidative stress condition. Detailed genetic and gene expression studies suggest that the *mir-60* loss-induced enhanced resistance against oxidative stress is mediated by activating the endocytosis machinery and downstream changes in expression of genes involved in the maintenance of cellular homeostasis, including those encoding lysosomal proteases. Further genetic studies suggest that *zip-10*, which encodes a bZIP transcription factor functioning in the innate immunity, serves as a key player in the adaptive response to oxidative stress induced by the loss of *mir-60*. Our findings provide new insights into the role of endocytotic processes and the innate immune system in an adaptive response against chronic oxidative stress.

## RESULTS

### miR-60 is exclusively expressed in the intestine and displays an age-associated decrease in expression

**Figure 1:**
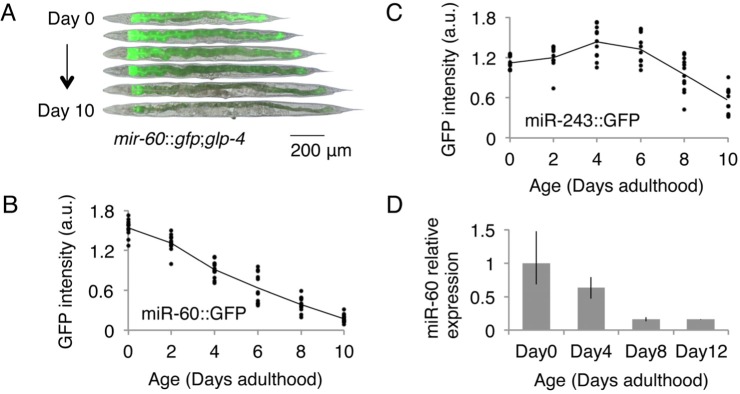
*C. elegans* miR-60 shows a specific spatio-temporal expression pattern (**A**) Fluorescent signals from miR-60::GFP, which are exclusively localized in the intestine, are shown. Approximately 25 individuals of (**B**) *mir-60::gfp*;*glp-4* and (**C**) *mir-243::gfp*;*glp-4* strains each were used for measuring GFP intensity at each time point examined, and the measurement for each individual is represented by a dot. Both strains were cultured at a restrictive temperature 23.5°C to induce germline deficiency. a.u. denotes arbitrary unit. Representative images of the *mir-60::gfp*;*glp-4* strain, which are close to the average signal intensity, are shown in (**A**). (**D**) A bar graph represents the relative expression of mature miR-60 (miR-60-3p strand) during normal aging in temperature-sensitive sterile mutants *spe-9*(*hc88*) cultured at 23.5°C (see Supplemental Text 2 for *spe-9* mutants). Error bars represent the range in the results of 2 biological replicates, in which the total RNA was purified from 2 independent experimental trials.

The *C. elegans* intestine, which is a counterpart to the gut, liver and adipose tissues in vertebrates, modulates energy metabolism and mediates the defense response against stress [31, 32]. We expected that miRNAs expressed in the intestine might contribute to the aging process by controlling genes involved in homeostatic maintenance. Of the approximately 100 miRNAs that were previously characterized for their spatio-temporal expression patterns, 3 miRNAs, including miR-60, are known to be expressed almost exclusively in the intestine (Fig 1A, [33]). To test the possible contribution of such intestinal miRNAs to the aging process, we first examined their expression changes throughout the lifetime of the animals. We used transgenic animals carrying constructs of the miRNA promoter fused to a green fluorescent protein (GFP) marker gene. In this analysis, we utilized a temperature-sensitive germline-less background, *glp-4*(*bn2*) [34], to avoid the effect of presence of gonads and embryos in parental bodies on microscopic quantifications. We found that miR-60::GFP shows an age-associated decrease in expression (Fig 1A,B). Conversely, another intestinal miRNA, miR-243, has a more stable expression change during aging (Fig 1C; we further identified that loss of *mir-243* does not affect lifespan (see Supplemental Fig 1A)), and so we focused specifically on *mir-60* in this study. We next examined the level of endogenous miR-60 using quantitative RT-PCR (qRT-PCR), and confirmed its continuous reduction during normal aging (Fig 1D), which is consistent with a recent report [35]. These observations imply a possible role of miR-60 in lifespan determination in *C. elegans*.

### Loss of *mir-60* results in increased resistance to a mild and long-term oxidative stress exposure

To directly test the importance of miR-60 in the aging process, we examined lifespans of mutants that completely lack the *mir-60* gene (*mir-60*(*n4947*)). Since we expected that a miRNA deletion may cause only a subtle effect on lifespan under a normal culture condition because of a fine-tuning capability of miRNAs, in this study we initially examined lifespans under stress conditions, which provide a metabolically sensitized background. Oxidative stress, which is a consequence of an imbalance between production and detoxification of reactive oxygen species (ROS), causes damage to biomolecules and tissues, accelerating the aging process [1]. Paraquat (PQ), an organic herbicidal compound, induces oxidative stress by generating superoxide from oxygen. PQ is widely used for the study of the oxidative stress response in *C. elegans*, where it is commonly used at concentrations of 100-200 mM for short-term exposure (0.5-several hours; [36-38]). In this study, however, we used much lower doses of PQ to determine the role of miRNAs in the long-term adaptive response against oxidative stress during aging.

**Figure 2:**
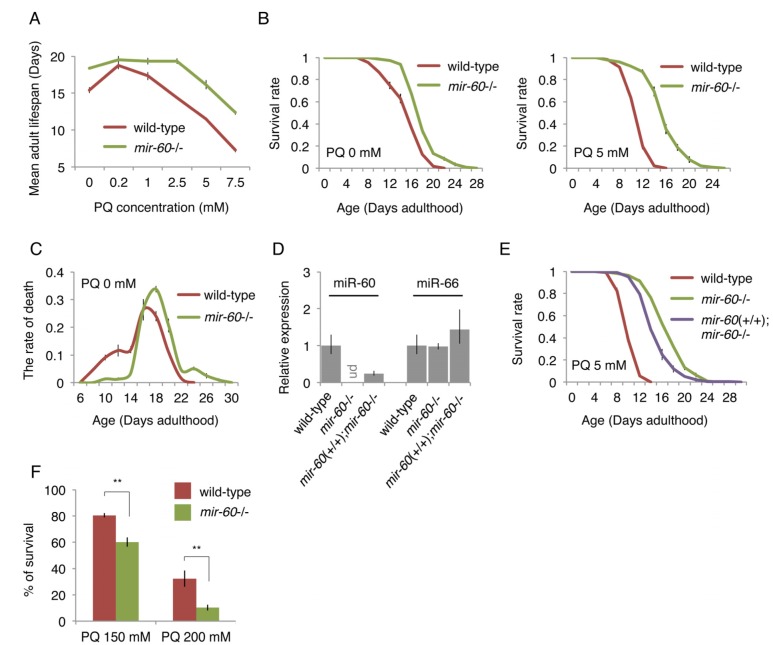
The *mir-60* loss dramatically extends lifespan under mild and long-term oxidative stress conditions (**A**) Mean adult lifespans of wild-type and *mir-60* mutant animals treated with different PQ concentrations are shown. (**B**) Survival curves of wild-type and *mir-60* animals examined under PQ 0 and 5 mM are shown. (**C**) Distribution of lifespans under the normal condition – the rate of death at each day examined – is shown. (**D**) The levels of miR-60 examined by qRT-PCR in the transgenics having the *mir-60* fragment (*mir-60*(+/+);*mir-60*−/−) and the control lines are shown. ‘ud’ denotes undetectable. The levels of another miRNA miR-66, which is known to be expressed constitutively [87], were also tested as a reference. Error bars represent SE calculated from the results of 3 independent trials of sample preparation. (**E**) Survival curves of the *mir-60* transgenics and control lines, which were treated with PQ 5 mM, are shown. The detailed lifespan assay results for (**A**-**C**) and (**E**) are available in Supplemental Figs 1B and 1D, respectively. All lifespan assays shown in this figure were performed at a standard temperature 20°C. Error bars on the survival curves represent SE calculated from 3-4 replicates. (**F**) Survival rate of wild-type and *mir-60* mutant animals treated with PQ 150 and 200 mM is shown. The assays were performed at 20°C. Error bars represent SE calculated from 4 replicates. P-values were calculated by unpaired t-test: **p<0.01.

We first assessed the impact of different PQ concentrations on lifespan in order to determine its optimal dose for a longitudinal survival study. Wild-type *C. elegans* animals were exposed to PQ 0 to 7.5 mM on solid media when they reached the young adult stage (referred to as Day 0 adulthood), and then their survival until death was scored, similar to conventional lifespan assays. We observed that wild-type animals treated with concentrations of PQ 2.5 mM or higher display significantly shortened adult lifespans, compared with those cultured under the normal condition of PQ 0 mM (Fig 2A). After multiple trials, we settled on PQ 5 mM for long-term oxidative stress exposure in this study, which is consistent with a recent report [39].

We next examined lifespans of mutants lacking the *mir-60* gene, and found that they show a dramatic lifespan extension under a wide range of PQ concentrations, including PQ 5 mM (Fig 2A and 2B right). In addition, we found that *mir-60* mutants have a slightly, but significantly extended lifespan under the normal culture condition of PQ 0 mM (Fig 2B left), and that this longevity benefit seems to be conferred predominantly by preventing early death at around Day 10 (Fig 2C). The detailed lifespan assay results, including numerical values and statistics, for Fig 2A-C are available in Supplemental Fig 1B. Notably, the lifespan extension observed in *mir-60* mutants treated with PQ was much larger than that seen with the untreated condition (approximately 40% and 70% lifespan extension under PQ 5 and 7.5 mM, respectively, while 19% lifespan extension under PQ 0 mM, compared to wild-type controls in each condition; Fig 2A and Supplemental Fig 1B). These observations support the idea that increased resistance to oxidative stress is a primary cause of the longevity phenotype seen in *mir-60* mutants.

To validate that the enhanced oxidative stress resistance observed in *mir-60* mutants is indeed caused by the deletion of the *mir-60* gene, we utilized a technique called MosSCI (for Mos1-mediated Single Copy Insertion) to insert a single copy gene [40], and established a transgenic line carrying the *mir-60* locus, which was crossed into the background lacking the endogenous *mir-60* gene (represented as *mir-60*(+/+);*mir-60*−/−, where *mir-60*(+/+) denotes the *mir-60* transgene in the homozygous state). We observed by qRT-PCR that miR-60 was undetectable in *mir-60* mutants, while its expression was partially recovered in the *mir-60*(+/+);*mir-60*−/− transgenic line (Fig 2D). Consistently, longer lifespans observed in *mir-60* mutants were partially suppressed in the transgenic line (Fig 2E). These results confirm that the *mir-60* deletion itself contributes to increased oxidative stress resistance and longevity.

In addition to the mild and long-term oxidative stress condition (i.e. the treatment with PQ 2.5-7.5 mM during adulthood), we investigated whether the *mir-60* loss can increase resistance to a transient and higher level of oxidative stress as well. Day 0 young adult animals were exposed to PQ at 150 and 200 mM for 6 hours in M9 buffer, and we examined their survival after 24 hours. In contrast to the dramatic resistance against the long-term mild PQ treatments, *mir-60* mutants did not show any increase in survival against the transient strong PQ treatments (Fig 2F). Rather, they seem to be slightly more sensitive to the higher doses of PQ. These observations suggest that the mir-60 loss-induced increased resistance against oxidative stress is conferred by an adaptive response rather than an acute response to stress.

### *mir-60* mutants show several common features of long-lived mutants

**Figure 3:**
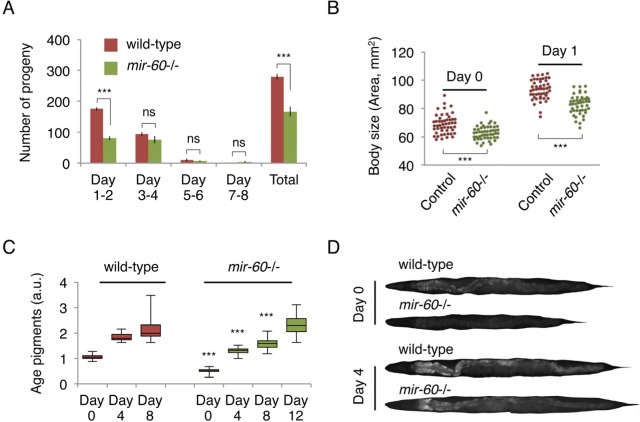
*mir-60* mutants exhibit features that are commonly observed in long-lived mutants (**A**) The number of progeny derived from wild-type and *mir-60* mutant parents was examined. Error bars represent SE calculated from the results of 8 individual parents in each strain. P-values were calculated by unpaired t-test: ***p<0.001, and ‘ns’ means not significant with *p*=0.1 or higher. The experiments were repeated 4 times, including 25 parents in total for each strain, and essentially the same results were obtained (data not shown). (**B**) Body size was examined using microscopic images of approximately 50 individuals for each strain/day and shown as each dot. P-values were calculated by unpaired t-test: ***p<0.001. (**C**) The box plot represents the distribution of age pigments. a.u. denotes arbitrary unit. Approximately 25 individual animals were examined at each time point. Signal intensities were normalized by the body size in individual animals. Unpaired t-test was used to calculate p-values (compared to wild-type control at each day examined): ***p<0.001. In this assay, animals were treated with PQ 5 mM during adulthood to enhance the effect of the *mir-60* loss on lifespan. Representative images having average pigment intensities are shown in (**D**) for Day 0 and Day 4 animals.

Lifespan extension is often positively correlated with the reduction of energy metabolism. Some ways to reduce energy expenditure include decreasing progeny production. Consistent with this idea, we found that *mir-60* mutants have fewer progeny, compared with wild-type animals, although the reproductive period itself was unaffected (Fig 3A). Furthermore, we noticed that *mir-60* mutants have a smaller body size compared with that in wild-type animals when they are young adult. To measure the body size of adult animals accurately, we utilized the germline-less *glp-4* background, analogous to Fig 1A-C. We found that *mir-60* mutants are indeed slightly smaller in body size, compared to that in control *glp-4* animals when they are young adult (Fig 3B). Additionally, we measured accumulation of age pigments –fluorescent compounds derived from metabolic by-products –, which is often used as a biomarker of aging [41, 42]. *mir-60* mutants show delayed onset of age pigment accumulation compared with wild-type animals (Fig 3C, D), reflecting the slower aging process in *mir-60* mutants.

### miR-60 does not function in the canonical longevity pathways

**Figure 4:**
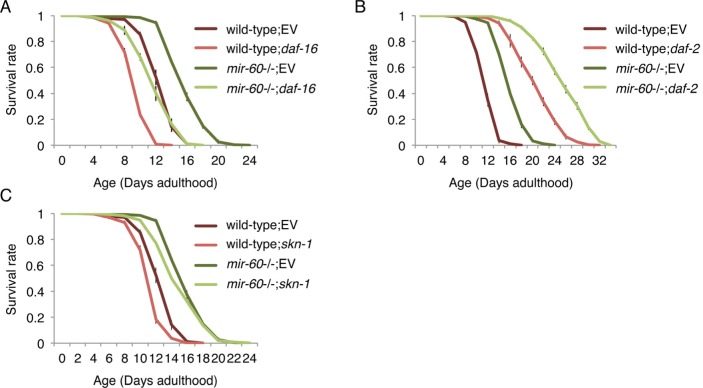
**The *mir-60* loss does not affect lifespans caused by RNAi inactivations against known aging genes**, including (**A**) *daf-16*, (**B**) *daf-2* and (**C**) *skn-1*. ‘EV’ denotes Empty Vector, L4440 plasmid DNA used as a control in feeding RNAi. All these lifespan assays were performed at 20°C under the PQ 5 mM condition. Error bars represent SE calculated from 3-4 replicates. The detailed results are available in Supplemental Fig 2A-C.

To understand the biological role of miR-60 in the adaptive response to long-term and mild oxidative stress, we investigated whether *mir-60* loss-induced longer lifespan under the PQ condition depends on known longevity factors. *C. elegans* DAF-16, a homolog of mammalian FOXO3a transcription factor, serves as a master regulator of stress responses and longevity, and is required for lifespan extension by the insulin-like signaling and the germline pathways as well as in some contexts of dietary restriction [2, 4, 43, 44]. We examined lifespan of *mir-60* mutants under the PQ 5 mM condition which were treated with RNA interference (RNAi)-mediated gene inactivation against *daf-16*, and found that lifespan reduction induced by *daf-16* RNAi in *mir-60* mutants was comparable to that in wild-type animals treated with the same RNAi (approximately 25% decrease in mean lifespans in both cases; Fig 4A). One trivial explanation is that this comparable lifespan reduction is due to insufficient RNAi inactivation of *daf-16*. To rule out the possibility, we used a null allele of *daf-16* (*daf-16*(*mgDf50*)) and obtained a similar comparable lifespan decrease in between *daf-16* single and *mir-60*;*daf-16* double mutants (Supplemental Fig 2A). This finding suggests that DAF-16 function is dispensable for the *mir-60* loss to promote adaptive response against the long-term mild oxidative stress.

In support of this conclusion, we found that *mir-60* mutants treated with *daf-2* RNAi have a longer lifespan than wild-type animals treated with *daf-2* RNAi (Fig 4B). The result was further validated by a genetic loss-of-function mutant *daf-2*(*e1370*) (Supplemental Fig 2B). The *daf-2* gene, which encodes an insulin-like receptor in *C. elegans*, negatively regulates DAF-16 activity, and its inactivation extends lifespan in a DAF-16-dependent manner [2, 4, 43, 44]. Our observation that *daf-2* longevity is further extended by combining with the *mir-60* loss suggests that miR-60 functions independently from the DAF-2/DAF-16 insulin-signaling axis.

In addition to DAF-16, SKN-1, a homolog of the mammalian Nrf2 transcription factor, is also known as an important regulator of stress responses and longevity [38, 45]. Analogous to DAF-16, we investigated the importance of SKN-1, and found that SKN-1 is similarly dispensable for the *mir-60* loss to enhance the adaptive response against oxidative stress (Fig 4C and Supplemental Fig 2C). Taken together, we conclude that miR-60 does not function in the canonical longevity pathways.

### miR-60 seems to directly modulate the endocytosis machinery

**Figure 5:**
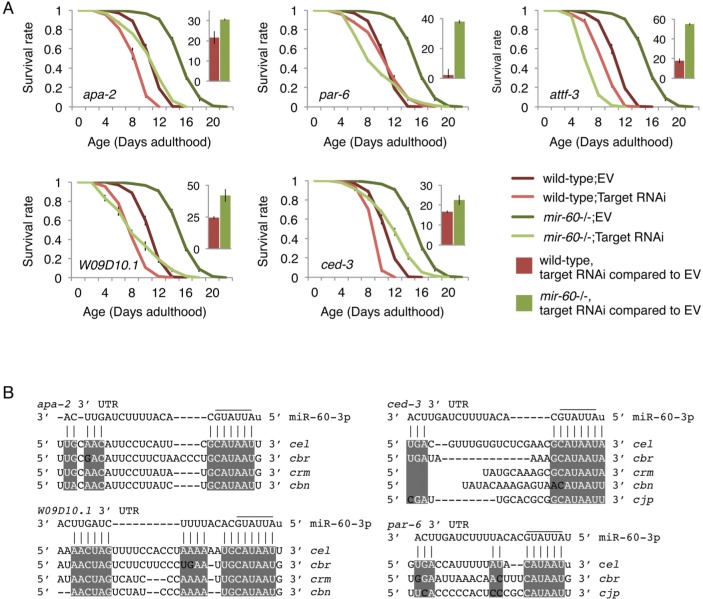
The *mir-60* loss-induced enhanced resistance against the long-term mild oxidative stress is significantly suppressed by RNAi inactivations against target candidates (**A**) Survival curves of wild-type and *mir-60* mutant animals treated with each RNAi are shown. ‘EV’ denotes Empty Vector control in feeding RNAi. Small bar graphs indicate the percentage of RNAi-induced lifespan reduction compared to the EV control for each strain. All these lifespan assays were performed at 20°C under the PQ 5 mM condition. Error bars represent SE calculated from 3-4 replicates. The detailed results are available in Supplemental Fig 3B. (**B**) Mature miR-60 strand (miR-60-3p) and its possible binding sites in 3′ UTR of target candidates are shown by vertical lines. Sequence alignments are based on TargetScan and RNAhybrid programs [46, 88]. Conserved regions among *C. elegans* (*cel*)-related species, including *C. briggsae* (*cbr*), *C. remanei* (*crm*), *C. brenneri* (*cbn*) and *C. japonia* (*cjp*), are highlighted by while-colored letters on gray backgrounds. Additional results are shown in Supplemental Fig 3C.

A better understanding of the biological role of miRNAs requires identification of their direct targets. In general, miRNAs negatively regulate their target gene activity[5], meaning that a phenotypic consequence caused by a miRNA deletion is mediated by increasing the activity of its target(s). We therefore hypothesized that *mir-60* loss-induced enhanced oxidative stress resistance would be suppressed by depletion of its target gene activity. Computational algorithms, including TargetScan[46], which predict miRNA targets based on 3′ UTR seed matches, were used to generate a list of miR-60 target candidates (Supplemental Table 1). We performed RNAi screens against the predicted targets to identify gene inactivations that significantly suppress the lifespan extension induced by the *mir-60* loss under the PQ 5 mM condition (see Supplemental Text 1). For potentially positive candidates identified from the screens, we performed conventional lifespan assays multiple times independently, and finally found that 9 RNAi clones reproducibly suppress the enhanced oxidative stress resistance induced by the *mir-60* loss (Fig 5A; additional results are shown in Supplemental Fig 3A). In all cases, the RNAi treatments significantly shorten the longer lifespans of *mir-60* mutants, com pared with those of wild-type control treated with the same RNAi. For example, RNAi inactivation against *apa-2* gene decreases the lifespans of wild-type and *mir-60* mutant animals by approximately 20% and 30%, respectively. In another example, while the *par-6* RNAi treatment is less effective to the wild-type lifespan, it completely suppresses the longer lifespan of *mir-60* mutants. Notably, of the 9 target candidates identified, 6 are involved in the endocytosis machinery, including APA-2/AP-2, PAR-6/PAR6, W09D10.1/ArfGAP, CAP-1/CAPZA, ATTF-3/AT hook, and PKC-3/aPKC ([47-49]; Supplemental Fig 4). All of these genes indeed have complementary sequences to the miR-60 seed region in their 3′ UTRs, which are highly conserved among related nematode species (Fig 5B and Supplemental Fig 3C). In addition, many of these are known to be expressed in the intestine (WormBase, http://www.wormbase.org/). These observations imply the possibility that miR-60 directly modulates genes functioning in intestinal endocytosis, contributing to the adaptive response against the long-term and mild oxidative stress in *C. elegans*.

### *mir-60* loss alters expression of genes involved in proteolysis and cytoprotection, but not expression of typical stress-responsive genes

**Figure 6:**
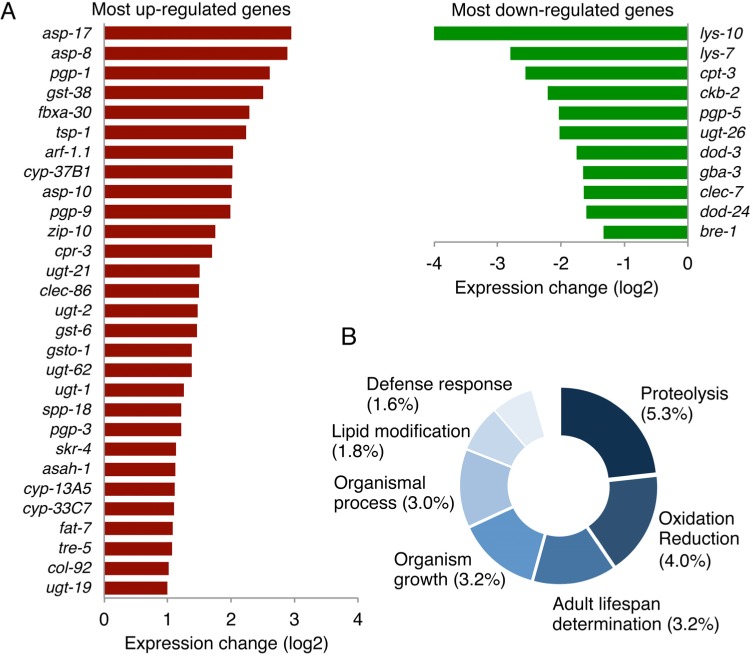
The loss of *mir-60* causes changes in expression of genes involved in proteolysis and cytoprotection (**A**) A list of genes significantly up/down-regulated by the *mir-60* loss is shown. Genes with lower expression levels and less functional annotation are omitted in this figure for space limitation. A full list of gene expression profile is available in Supplemental Tables 3 and 5. (**B**) The result of GO-based GSEA is shown. Percentages represent the rate of gene count classified into each functional category compared to the total gene count examined. The detailed result, including statistics, is available in Supplemental Table 4.

To further explore the downstream effect caused by the *mir-60* loss, we performed a transcriptome analysis using mRNA sequencing. In this study, we used *spe-9*(*hc88*), a temperature-sensitive sterile strain[50], which has been shown in previous studies to have a wild-type-like lifespan and widely used in gene expression studies to reduce the effect of RNA contamination from younger progenies[12, 51-53]. We prepared total RNA from *mir-60*;*spe-9* double mutants and control *spe-9* single mutants at Day 0 young adult stage and used it for cDNA library construction. In addition to Day 0 adulthood, total RNA was also isolated from 50% survival time point (see Supplemental Text 2). The libraries established were then examined by next-generation sequencing (processed data is available in Supplemental Table 2, and additional data, including the summary of sequencing reads, reproducibility check and confirmation of gene expression by qRT-PCR, are shown in Supplemental Fig 5A-D). We found that 120 genes were up-regulated and 27 genes were down-regulated significantly by >2-fold each in the *mir-60* loss background (Fig 6A and Supplemental Table 3).

To explore the contribution of the *mir-60* loss-induced gene expression changes to the longevity benefit, we next performed gene set enrichment analysis. In this analysis, 520 genes with statistically significant >1.5-fold changes in expression – of those, 437 are up-regulated and 83 are down-regulated – were used as an input to increase the chance of identifying their common biological function. Using gene ontology (GO) term-based functional annotation clustering [48, 54, 55], we found that a significant portion of the genes whose expression is significantly altered by the *mir-60* loss are involved in proteolysis, oxidation reduction, lifespan determination and defense response (Fig 6B and Supplemental Table 4). For example, genes encoding ASpartyl family proteases (ASP), which catalyze the proteolytic process, were increased in expression in the *mir-60* loss background (Fig 6A). Three up-regulated Asp genes, including *asp-17*, *asp-8* and *asp-10*, are orthologs of human lysosomal aspartic cathepsin D gene, which generally function in the endocytic pathway, where they play multiple roles, including protein degradation/processing and turnover of organelle[56]. Other important gene sets up-regulated by the *mir-60* loss are those encoding glutathione S-transferase (GST, e.g. *gst-38*), UDP glucuronosyl-transferase (UGT, e.g. *ugt-21*) and cytochrome P450 (CYP, e.g. *cyp-13A5*) (Fig 6A). These three gene groups act together in xenobiotic metabolism[57], suggesting that the *mir-60* loss induces the detoxification system, improving cellular maintenance.

In contrast to these expression changes, typical stress responsive genes, such as heat shock protein genes of *hsp-16* families and antioxidant genes, including a superoxide dismutase gene *sod-3* and a catalase gene *ctl-3*, which are the most DAF-16/FOXO-responsive factors [58, 59], and also *gst-4*, which is directly regulated by SKN-1[60, 61], were not affected in the *mir-60* loss background (Supplemental Table 3). This is consistent with our conclusion that *mir-60* loss-induced enhanced resistance against long-term mild oxidative stress is not mediated by DAF-16 or SKN-1 (Fig 4).

It is also important to note that the expression profile observed in *mir-60* mutants have some overlapping features with that observed after prolonged exposure to a toxic heavy metal, cadmium[62]. More specifically, the *Asp* genes and P-glycoprotein genes e.g. *pgp*-*1/9*, and some Gst genes, including *gst-38*, are up-regulated, while lysozyme genes (*Lys*), such as *lys-10*, are down-regulated commonly in Cui et al. and our study. More importantly, many of those commonly changed genes are predominantly induced following prolonged cadmium exposure but not by a short-term cadmium exposure[62]. Altogether, the *mir-60* loss-induced enhanced oxidative stress resistance is not caused by a typical stress response, rather mediated by an adaptive mechanism that possibly involves the maintenance of cellular homeostasis.

### Inactivation of *zip-10* disrupts *mir-60* loss-induced adaptive response against oxidative stress

**Figure 7:**
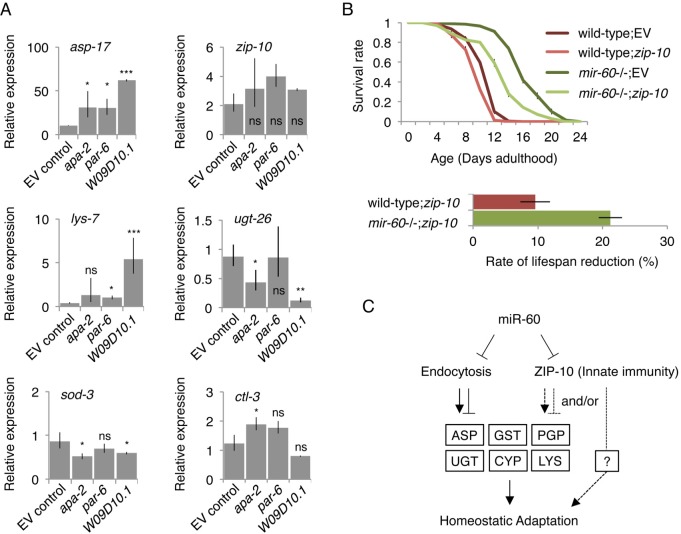
Inhibiting miR-60 target candidates abolish the *mir-60* loss-induced gene expression changes and adaptive response against oxidative stress (**A**) Expression was examined by qRT-PCR in the *mir-60*;*spe-9* double mutant background, and the results were standardized by the expression level in the control *spe-9* animals exposed to the empty vector control in RNAi. Error bars represent SE and p-values were calculated by paired t-test based on 3 independent trials of sample preparation: *p<0.1; **p<0.01; ***p<0.001. (**B**) Survival curves of wild-type and *mir-60* mutant animals exposed to *zip-10* RNAi are shown. A small bar graph below represents the rate of lifespan reduction of *zip-10* RNAi-treated animals compared to those treated with the control RNAi. Error bars represent SE calculated from 3 replicates. The detailed results are available in Supplemental Fig 6. (**C**) A model illustrates the mechanism underlying *mir-60* loss-induced adaptive response against chronic oxidative stress. Arrows and blunt arrows denote positive and negative interactions, respectively. miR-60 appears to directly modulate the activity of endocytosis machinery, which regulates downstream expression of genes, such as those encoding Aspartyl protease (ASP), P-glycoprotein (PGP), glutathione S-transferase (GST), UDP glucurono-syltransferase (UGT), cytochrome P450 (CYP) and lysozyme (LYS). ZIP-10, which also seems to be directly controlled by miR-60, promotes homeostatic adaptation possibly through *mir-60* loss-responsive factors such as PGP, and/or unidentified factor(s) (shown by broken lines).

Next, to investigate the relationship between the *mir-60* loss-induced gene expression changes and the target candidates of miR-60, we examined expression of the *mir-60* loss-regulated genes in the backgrounds of RNAi inactivation of endocytosis-related genes, including *apa-2*, *par-6* and *W09D10.1*. We found several patterns of changes; for example, one of the Asp genes, *asp-17*, which is up-regulated in *mir-60* mutants, showed a further dramatic increase in expression, while *zip-10* was essentially not affected in all these RNAi knockdowns (Fig 7A; see below for *zip-10*). In another example, *lys-7*, which is down-regulated by the *mir-60* loss, is rather up-regulated by *W09D10.1* inactivation. Also, *ugt-26* was further down-regulated by *W09D10.1* inactivation. It seems likely that the endocytosis deficiencies disrupt the balanced expression of these *mir-60* loss-induced genes. In contrast to those changes, genes unaffected by the *mir-60* loss, such as DAF-16-responsive genes *sod-3* and *ctl-3*, were not substantially changed by the inactivation of endocytosis-related genes (Fig 7A). These observations suggest that the *mir-60* loss causes coordinated and specific alterations of gene expression that are important for adaptive response against the long-term mild oxidative stress.

To further explore the relationship between the *mir-60* loss-induced gene expression changes and the adaptive response against oxidative stress, we examined the effect of inhibiting the *mir-60* loss-induced genes on lifespan under oxidative stress conditions. We hypothesized that inactivation of genes up-regulated by the *mir-60* loss would disrupt the longevity effect if they function downstream of miR-60. We found that for many of the up-regulated genes we examined, RNAi knockdown slightly but significantly shortened wild-type lifespan under the PQ 5 mM condition (Supplemental Fig 6). Of those, RNAi against *zip-10*, which belongs to a conserved bZIP transcription factor family, significantly reduced the *mir-60* loss-induced lifespan extension, compared with wild-type animals treated with the same RNAi (Fig 7B). *zip-10*, a homolog of human BATF3 gene, is known to be expressed in the adult intestine [63], and up-regulated following exposure to a bacterial pathogen in *C. elegans,* suggesting its role in innate immunity [64].

It is plausible that the innate immune system contributes to the adaptive response against oxidative stress in *mir-60* mutants. We found that in addition to *Asp*/*Gst*/*Ugt*/*Cyp* genes above, P-glycoprotein (Pgp) genes, including *pgp-1*, *pgp-3* and *pgp-9*, which encode ATP-binding membrane transporters involved in pathogen defense responses [65, 66], are up-regulated by *mir-60* loss (Fig 6A). Pgps are known to act as energy-dependent drug efflux pumps to extrude xenobiotic compounds [67], protecting cells from toxins, including those generated from pathogens. The *mir-60* loss-induced increased expression of *zip-10* may contribute to the maintenance of cellular homeostasis by regulating its downstream genes, such as *Pgp*s (Fig 7C). Importantly, we noticed that *zip-10* is also one of the predicted targets of miR-60 (Supplemental Fig 3C), suggesting that miR-60 directly regulates *zip-10* as well as the endocytosis components, consistent with the observation that inactivations of endocytosis-related genes do not affect *zip-10* expression (Fig 7A). Taken together, the endocytotic and *zip-10*-mediated innate immune systems, which are directly controlled by miR-60, coordinate expression of genes involved in the cellular homeostasis, promoting adaptive response against the long-term mild oxidative stress (Fig 7C).

## DISCUSSION

Animals are constantly exposed to potential risks that prevent a normal lifespan, such as starvation, temperature changes and oxidative stress. In addition to ROS as a natural by-product in normal energy metabolism, ROS are also generated from exposure to radiation, pollutants and pathogens, causing chronic oxidative stress and gradually accumulating damage in cells and tissues during aging. In this study, we show that *C. elegans* miRNA miR-60, which is exclusively expressed in the intestinal tissue, modulates an adaptive response against mild and long-term oxidative stress exposure. It seems that this adaptive process is achieved by ensuring the maintenance of cellular homeostasis through the endocytosis and innate immune pathways.

Our genetic studies combined with computational miRNA target prediction have identified the target candidates of miR-60, which include components of the endocytosis machinery. Of those, AP-2, PAR6 and ArfGAP, are generally known to function in the endocytic recycling pathway, which allow cells to reuse endocytosed proteins and lipids[68, 69]. Internalized molecules are subjected to degradation in lysosomes or are recycled back to the cell surface. miR-60 might coordinate the balance between degradation and recycling depending on the cellular state. We also noticed that additional endocytosis factors are predicted as targets of miR-60, which include *rme-1*, *mtm-6* and *rabs-5* (Supplemental Fig 4), implying the possibility that many more genes related to the endocytosis are directly modulated by miR-60, fine-tuning the cellular maintenance in the intestine.

Beyond identifying the endocytic regulators as miR-60 targets, we also found that *ced-3* is one of the confirmed target candidates of miR-60; the RNAi inactivation significantly shortens *mir-60* loss-induced lifespan extension under the oxidative stress condition (Fig 5A). The *ced-3* gene encodes a caspase (for cysteine-aspartate protease) and is required for normal apoptosis activity[70]. A recent study has shown that loss-of-function mutations in the conserved intrinsic apoptosis-signaling pathway, including *ced-3*, suppress hormesis-induced longevity – a phenomenon whereby exposing animals to low levels of stress can trigger subsequent beneficial effects –, although the necessity of the apoptosis-signaling pathway for hormetic response is independent from inhibition of apoptosis[71]. We have observed that *mir-60* mutants treated with a much lower dose of PQ (0.2 mM) have a slightly but significantly longer lifespan than those cultured under the normal condition of PQ 0 mM (Supplemental Fig 1B/C). This implies that a *mir-60* loss-regulated downstream mechanism functions redundantly with the hormesis-induced pathway. Alternatively, a *mir-60* loss-induced change might optimize a hormesis effect, leading to a further prolonged lifespan. Although this needs to be investigated further, our observation may provide new insight into a role of apoptosis or caspase activity in an adaptive response against stress.

In addition to the miR-60 target candidates described above, we found that one of the bZip family transcription factor genes, *zip-10*, appears to be directly controlled by miR-60. As it has been reported previously that miRNAs do not often affect their target gene expression at the mRNA level – although this is still controversial subject –[5], we have observed that expression of the endocytosis genes and *ced-3* is not essentially changed by the *mir-60* loss in our RNA sequencing study (Supplemental Table 3). In contrast, *zip-10* was found to be significantly up-regulated by the *mir-60* loss (Fig 6A). We found that in addition to two miR-60 complementary sequences in the 3′ UTR of *zip-10*, there is also one possible miR-60 biding site in its exon region (Supplemental Fig 3C), implying a potential control of *zip-10* by miR-60 at both transcriptional and post-transcriptional levels. *C. elegans zip-10* and its human homolog BATF3 gene are both involved in the immune system, including their role in pathogen responses[64, 72]. Although the function of *zip-10* remains largely unexplored in *C. elegans*, its downstream molecular mechanism might overlap with those of human BATF3.

Lastly, while the target candidate and downstream genes of miR-60 are highly conserved among animal species, miRNAs having the same seed sequence as miR-60 are limited to the nematode and some insect species (Supplemental Fig 7A). However, we have found that miRNA genes having similar seed sequences, including those with a 1-bp mismatch or 1-bp shift, exist in the human genome, such as miR-544a and hsa-miR-4795-3p (Supplemental Fig 7B). It is possible that these miRNAs also regulate the endocytic activity in human. Indeed, for example, AP2B1, a human homolog of *C. elegans* APA-2, and its associated protein AAK1, that are both involved in the endocytosis, are predicted as targets of miR-544a by the TargetScan program[46]. In addition, TargetScan predicts BATF3 as a target of hsa-miR-4795-3p. These miRNAs might play an important role in an adaptive response against chronic oxidative stress in human. *C. elegans mir-60* mutants may serve as a model to deepen our understanding of a mechanism underlying long-term adaptive response against stress.

## MATERIALS AND METHODS

### *C. elegans* strains

*C. elegans* strains used in this study are summarized in Supplemental Table 1. *C. elegans* animals were cultured on solid nematode growth media (NGM) plates and handled with a standard technique[73]. All strains used for lifespan and stress survival assays were backcrossed extensively to our own wild-type N2 Bristol strain to remove background mutations and potential genetic variations. The temperature-sensitive strains, including animals in the *spe-9*(*hc88*) or *glp-4*(*bn2*) background, were maintained at a permissive temperature 15°C, and eggs prepared were placed at a restrictive temperature 23.5°C to induce sterility for lifespan/stress assays, microscopy or RNA isolation. All other strains were maintained at a standard temperature 20°C. PCR primers used for genotyping are summarized in Supplemental Table 7.

### Lifespan assays

We performed lifespan assays as previously described [12, 16] with some modifications. Briefly, *C. elegans* animals were maintained and grown continuously at 20°C on NGM plates seeded with *E. coli* OP50 for at least 4 generations (about for 2 weeks at 20°C) before doing egg preparation for lifespan assays. This procedure is to erase a potential effect of starvation/diapause on gene expression and lifespan [74, 75]. For growth synchronization, eggs were prepared by a standard bleach/NaOH treatment and directly placed on OP50- or RNAi bacteria-seeded NGM plates, and cultured until they reached the young adult stage (78-80 hours and 68-70 hours from embryo at 20°C and 23.5°C, respectively). 5-fluorodeoxyuridine (FUDR; a DNA replication inhibitor, Sigma-Aldrich) was added to plates at 325 μM in the final concentration to prevent growth of progeny. The date of adding FUDR was defined as Day 0 adulthood. We checked the survival of animals every other day, and scored as dead when they no longer responded to a gentle touching with a platinum wire. Animals that died unnaturally during assays (vulval bursting, internal hatching of eggs) were excluded from calculations. Approximately 100 animals were tested on each plate with 3-4 replicates. P-values were calculated by log-rank test using results merging all 3-4 replicates in each trial. All lifespan assays were repeated at least three times independently, including 3-4 replicates in each trial, and one of the representative trials is shown. We further performed lifespan assays of *mir-60* mutants under a non-FUDR condition to exclude a possible effect of FUDR on lifespan, and confirmed that loss of *mir-60* indeed increases oxidative stress resistance and lifespan (see Supplemental Fig 1E).

### Paraquat treatment

Methyl viologen dichloride (Paraquat/PQ; Sigma-Aldrich) was used a source of ROS. For long-term PQ treatment, staged Day 0 young adult animals cultured under normal conditions were exposed to PQ at final concentrations of 0.1-7.5 mM on standard solid NGM plates seeded with OP50 or RNAi bacteria, and we examined their survival until death. For short-term PQ treatment, staged Day 0 adult or L4 stage animals were incubated in M9 buffer containing 150 or 200 mM PQ with rotation for 6 hours at 20°C. After incubation, treated animals were washed 3 times with M9 buffer and placed on OP50-seeded solid NGM plates and recovered at 20°C, and then we examined their survival 24 hours post treatment.

### RNAi gene inactivation

*C. elegans* animals were exposed to freshly cultured RNAi bacteria from embryos with a standard feeding RNAi method[76], and placed at an appropriate temperature condition, 20°C or 23.5°C depending on the strain background used. Most of the RNAi clones used in this study were derived from Ahringer's library [77], and all clones with a positive effect were confirmed by DNA sequencing.

### Establishment of a *mir-60* rescue line by MosSCI

A *mir-60* locus, which encompasses 1.1 kb upstream (up to an adjacent gene) and 1 kb downstream genomic region of *mir-60* precursor, was amplified by PCR and subcloned into pCFJ352, a MosSCI targeting vector[40]. After DNA sequence confirmation, the plasmid DNA was injected with other control plasmid DNAs into a MosSCI *C. elegans* strain (*ttTi4348*;*unc-119*(*ed3*)), which is originally derived from EG6701 strain. A transgenic line having the entire rescue fragment was backcrossed twice into our wild-type N2 strain, and then crossed into the *mir-60* loss background. The original *unc-119* mutation was removed by backcrossing. Primers used are summarized in Supplemental Table 7.

### Quantitative microscopy

GFP signal intensity, body size and autofluorescence accumulation were measured by microscopic observations (Leica DM6000B or M205FA). Images were obtained from a whole animal body individually with the same microscopic settings (e.g. magnification, exposure time) with a focus on the center of each animal based on its pharynx and/or vulva. *C. elegans* images were quantified by ImageJ [78] and straightened for presentation purpose after quantification. For the body size analysis, the growth of *mir-60*;*glp-4* double mutants and *glp-4* control animals was synchronized through the 1st larval stage (L1) arrest to reduce the growth variation among animals caused by different hatching timing, and those animals were cultured at 23.5°C. For measuring autofluorescence accumulation, age pigments were visualized and quantified by fluorescence microscope with a GFP filter set (Excitation: 480/40 nm; Barrier filter: 510LP).

### RNA isolation

Animals collected were first washed with M9 buffer 3 times, then incubated in M9 buffer with rotation for 30 minutes to allow them to digest bacteria within the intestine. Total RNAs were purified using *mirVana* miRNA Isolation Kit or Trizol (Ambion/Life Technologies) combined with RNA Clean & Concentrator (ZYMO Research), according to the manufacturers’ instructions.

### Gene expression profiling

Total RNA was isolated from *mir-60;spe-9* double mutants and *spe-9* control animals when they were Day 0 young adult and also reached 50% survival time points (see Supplemental Text 2 for details). cDNA libraries for RNA sequencing were established from 4 μg of the total RNA for each using TruSeq Stranded mRNA Sample Prep Kit (Illumina) with indexed adapters, according to the manufacturer's instruction. The libraries were quantified using NEBNext Library Quant Kit (New England Biolabs) on a real-time PCR instrument 7900HT (Applied Biosystems), and sequenced on the Illumina HiSeq platform with 100 bp single end options at Australian Genome Research Facility Ltd (www.agrf.org.au). Sequencing reads were aligned to the *C. elegans* genome (WS220) using Bowtiew program (version 2.1.0)[79] then incorporated into TopHat program (version 2.1.0)[80]. Expression levels of genes were calculated using Cufflinks software (version 2.2.1)[81], and were represented as fragment per kb per million reads (FPKM; Supplemental Table 2). The DEseq program[82] was also used to list genes with differential expression between those two strains at Day 0 young adult for which the biological replicates are available (Supplemental Table 3). The number of gene count in all samples examined is summarized in Supplemental Table 5. Gene enrichment analysis was performed using DAVID Functional Annotation Tool [55] for differentially expressed genes between two samples (Supplemental Table 4).

### qRT-PCR

qRT-PCR was performed to investigate expression of mature miR-60 (miR-60-3p) and coding genes with the Universal ProbeLibrary technology (UPL; Roche) on 7900HT or StepOne instrument (Applied Biosystems). cDNAs for the miR-60 were synthesized using a hairpin-loop adapter[83, 84] with ProtoScript II reverse transcriptase (New England Biolabs), and cDNAs for coding genes were synthesized using random hexamers. The cDNA libraries established were diluted with water and then used as a template in qPCR reaction with a UPL probe and primers specific to each gene examined. All primer sequences and UPL probes used in this study are summarized in Supplemental Table 7. The results were analyzed by the delta-delta Ct method[85] and normalized by the average of 3 control genes, including *ama-1*, *cdc-42* and *pmp-3*, which have been shown to have a stable expression pattern in *C. elegans* aging mutants[86]; all these 3 genes were indeed found to be expressed at similar levels between the control and *mir-60* mutant animals (see Supplemental Table 3). P-values were calculated by paired t-test from delta Ct values between two samples compared, which were obtained from 3 biological replicates, in which the total RNA was purified from 3 independent trials.

### Accession number

Raw sequencing reads from Illumina HiSeq and processed data have been deposited in the NCBI Gene Expression Omnibus (GEO) with the accession number GSE83239.

## SUPPLEMENTAL FIGURES AND TABLES
















