# Supplementary material for: An intestinal microRNA modulates the homeostatic adaptation to chronic oxidative stress in C. elegans
Source: Aging (Albany NY). 2016 Sep 12;8(9):1979–96. doi: 10.18632/aging.101029 (PMC5076448; doi:10.18632/aging.101029)
Supplement: Supplementary file 1 [file aging-08-1979-s001.pdf]

SUPPLEMENTAL FIGURES

A

wild-type

PQ concentration	0 mM	0.2 mM	1.0 mM	2.5 mM	5.0 mM	7.5 mM
Number of replicate	4	4	4	3	3	3
Total number of dead worms	329	304	307	232	253	184
Mean adult lifespan+/-SEM (Days)	15.4+/-0.4	18.7+/-0.5	17.3+/-0.6	14.4+/-0.0	11.5+/-0.1	7.3+/-0.3
Median, 25th, 75th percentiles (Days)	16, 12, 18	18, 16, 20.5	18, 16, 19	14, 12, 16	12, 10, 12	8, 6, 8
P-value compared to PQ 0 mM	-	4.5E-21	9.1E-07	0.0010	2.5E-35	6.0E-93

mir-60 -/-

PQ concentration	0 mM	0.2 mM	1.0 mM	2.5 mM	5.0 mM	7.5 mM
Number of replicate	4	3	4	3	3	3
Total number of dead worms	324	257	303	231	210	248
Mean adult lifespan+/-SEM (Days)	18.3+/-0.2	19.5+/-0.7	19.3+/-0.4	19.3+/-0.4	16.1+/-0.6	12.4+/-0.3
Median, 25th, 75th percentiles (Days)	18, 16, 20	18, 18, 22	20, 18, 22	20, 16, 22	16, 14, 18	14, 12, 14
P-value compared to PQ 0 mM	-	0.00058	0.0010	0.0021	4.1E-07	3.5E-85
P-value compared to wild-type each PQ	6.6E-15	0.0125	1.3E-12	3.3E-29	2.3E-34	2.2E-53

B

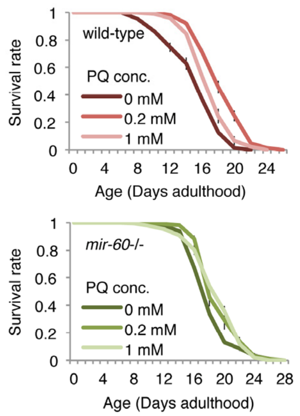

C

PQ 0 mM

Strain/Genotype	wild-type	*mir-60-/-*	*mir-60(+/+);mir-60-/-*
Number of replicate	3	3	3
Total number of dead worms	347	504	356
Mean adult lifespan+/-SEM	15.6+/-0.7	19.9+/-0.1	18.6+/-0.0
P-value compared to *mir-60-/-*	3.6E-31	-	0.0157

PQ 5 mM

Strain/Genotype	wild-type	*mir-60-/-*	*mir-60(+/+);mir-60-/-*
Number of replicate	3	3	3
Total number of dead worms	294	426	318
Mean adult lifespan+/-SEM	10.5+/-0.2	17.3+/-0.1	15.3+/-0.5
P-value compared to *mir-60-/-*	5.5E-91	-	2.1E-10

PQ 7.5 mM

Strain/Genotype	wild-type	*mir-60-/-*	*mir-60(+/+);mir-60-/-*
Number of replicate	3	3	3
Total number of dead worms	278	412	316
Mean adult lifespan+/-SEM	7.5+/-0.3	13.5+/-0.3	11.5+/-0.3
P-value compared to *mir-60-/-*	4.8E-98	-	1.4E-15

D

PQ 0 mM

Strain/Genotype	wild-type	*mir-60-/-*	*mir-243-/-*
Number of replicate	4	4	4
Total number of dead worms	303	320	438
Mean adult lifespan+/-SEM	14.8+/-0.1	17.7+/-0.5	15.3+/-0.4
P-value (compared to wild-type)	-	3.7E-10	0.317

PQ 5 mM

Strain/Genotype	wild-type	*mir-60-/-*	*mir-243-/-*
Number of replicate	3	3	3
Total number of dead worms	313	405	216
Mean adult lifespan+/-SEM	10.3+/-0.3	15.4+/-0.7	10.4+/-0.5
P-value (compared to wild-type)	-	8.0E-64	0.590

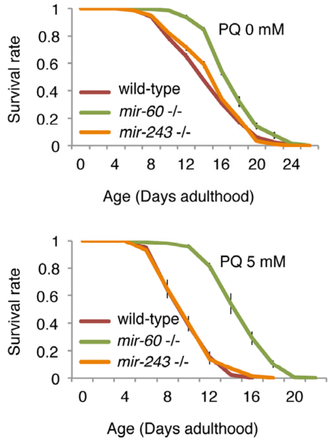

E

No FUDR, PQ 0 mM, 20 °C

Strain Name	wild-type	*mir-60-/-*
Total number of dead worms	148	133
Mean adult lifespan+/-SD	13.1+/-2.7	14.4+/-3.1
P-value compared to wild-type	-	0.00145

No FUDR, *spe-9*(*hc88*), 23.5 °C

	PQ 0 mM		PQ 5 mM	
Strain Name	*spe-9-/-*	*mir-60-/-;spe-9-/-*	*spe-9-/-*	*mir-60-/-;spe-9-/-*
Total number of dead worms	263	236	203	254
Mean adult lifespan+/-SD	6.4+/-1.4	7.2+/-1.6	4.4+/-0.8	6.0+/-1.3
P-value compared to *spe-9* control	-	9.8E-06	-	3.7E-23

Supplemental Figure 1. The *mir-60* loss increases resistance against a mild and long-term oxidative stress exposure. (**A**) Unlike miR-60, another intestinally expressed miRNA, miR-243, seems not to be involved in lifespan determination and oxidative stress survival. (**B**) A table shows numerical values and statistics for the survival curves for Fig 2A-C. (**C**) Exposing animals to much lower dose of PQ 0.2 to 1.0 mM extends lifespan in both wild-type and *mir-60* mutant animals (see Discussion). (**D**) A table shows numerical values and statistics for the survival curves for Fig 2E. (**E**) Lifespan extension observed in *mir-60* mutants was confirmed under non-FUDR conditions. To exclude a possible effect of a DNA replication inhibitor FUDR on the *mir-60* loss-induced longevity, lifespan assays were performed without FUDR, where animals were individually transferred onto new plates everyday or every other day during the reproductive period as necessary. For the oxidative stress condition, as we found that the PQ 5 mM treatment causes an egg-laying defect – hatching of embryos inside of parental bodies at a higher frequency, we utilized a sterile background caused by a *spe-9*(*hc88*) mutation. We could confirm that the *mir-60* loss could extend lifespan under both normal and oxidative stress conditions independently from FUDR.

A **RNAi, PQ 5 mM condition; see Fig 4A for survival curves plotted**

Strain/RNAi	wild-type;EV	*mir-60-/-*;EV	wild-type;*daf-16* RNAi	*mir-60-/-*;*daf-16* RNAi
Number of replicate	4	4	3	3
Total number of dead worms	368	462	281	345
Mean adult lifespan+/-SEM	13.0+/-0.5	16.2+/-0.3	9.8+/-0.1	12.2+/-0.6
Lifespan reduction (%)	0+/-4.0	0+/-1.6	24.5+/-1.0	24.9+/-3.7

PQ 0 mM, *daf-16*(*mgDf50*)

Strain/Genotype	wild-type	*mir-60-/-*	*daf-16-/-*	*mir-60-/-*; *daf-16-/-*
Mean adult lifespan+/-SEM	15.1+/-0.3	16.6+/-0.3	11.1+/-0.1	13.1+/-0.4
Lifespan reduction (%)	0+/-2.0	0+/-2.0	26.1+/-0.4	21+/-2.6

PQ 5 mM, *daf-16*(*mgDf50*)

Strain/Genotype	wild-type	*mir-60-/-*	*daf-16-/-*	*mir-60-/-*; *daf-16-/-*
Mean adult lifespan+/-SEM	8.7+/-0.4	15.1+/-0.8	7.6+/-0.2	13.1+/-0.2
Lifespan reduction (%)	0+/-5.0	0+/-5.3	12.1+/-2.1	13.2+/-1.6

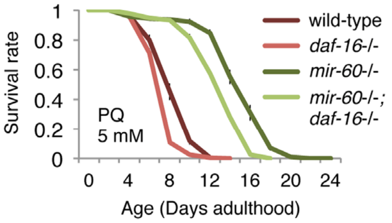

B **RNAi, PQ 5 mM condition; see Fig 4B for survival curves plotted**

Strain/RNAi	wild-type;EV	*mir-60-/-*;EV	wild-type;*daf-2* RNAi	*mir-60-/-*;*daf-2* RNAi
Number of replicate	4	4	4	4
Total number of dead worms	266	384	309	377
Mean adult lifespan+/-SEM	12.1+/-0.3	16.4+/-0.3	20.9+/-0.9	25.7+/-0.7

PQ 0 mM, *daf-2*(*e1370*)

Strain/Genotype	wild-type	*mir-60-/-*	*daf-2-/-*	*mir-60-/-*; *daf-2-/-*
Mean adult lifespan+/-SEM	16.0+/-0.3	17.8+/-0.2	54.9+/-1.0	56.9+/-3.4

PQ 5 mM, *daf-2*(*e1370*)

Strain/Genotype	wild-type	*mir-60-/-*	*daf-2-/-*	*mir-60-/-*; *daf-2-/-*
Mean adult lifespan+/-SEM	10.3+/-0.3	15.4+/-0.7	33.8+/-0.5	42.4+/-1.0

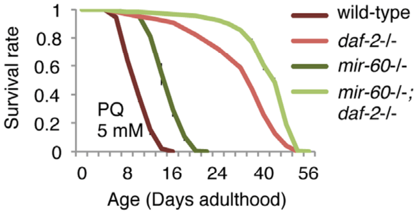

C **RNAi, PQ 5 mM condition; see Fig 4C for survival curves plotted**

Strain/RNAi	wild-type;EV	*mir-60-/-*;EV	wild-type;*skn-1* RNAi	*mir-60-/-*;*skn-1* RNAi
Number of replicate	4	4	4	4
Total number of dead worms	368	462	416	458
Mean adult lifespan+/-SEM	13.0+/-0.5	16.2+/-0.3	11.7+/-0.3	15.4+/-0.1
Lifespan reduction (%)	0+/-4.0	0+/-1.6	9.9+/-2.2	5.3+/-0.7

PQ 0 mM, *skn-1*(*zu135*)

Strain Name	wild-type	*mir-60-/-*	*skn-1-/-*	*mir-60-/-;skn-1-/-*
Mean adult lifespan+/-SEM	14.4+/-0.7	16.4+/-0.3	12.8+/-0.3	14.5+/-0.2
Lifespan reduction (%)	0+/-4.5	0+/-2.1	11.2+/-1.9	11.8+/-1.3

PQ 0 mM, *skn-1*(*zu67*)

Strain Name	wild-type	*mir-60-/-*	*skn-1-/-*	*mir-60-/-;skn-1-/-*
Mean adult lifespan+/-SEM	14.3+/-0.4	16.5+/-0.1	10.6+/-0.1	11.4+/-0.4
Lifespan reduction (%)	0+/-3.0	0+/-0.5	25.7+/-0.6	30.9+/-2.7

PQ 5 mM, *skn-1*(*zu135, zu67*)

			zu135		*zu67*	
Strain Name	wild-type	*mir-60-/-*	*skn-1-/-*	*mir-60-/-;skn-1-/-*	*skn-1-/-*	*mir-60-/-;skn-1-/-*
Mean adult lifespan+/-SEM	10.0+/-0.2	14.8+/-0.5	11.4+/-0.6	18.3+/-0.6	11.8+/-0.7	16.5+/-0.1
Lifespan reduction (%)	0+/-2.3	0+/-3.0	*-14.1+/-5.8*	*-23.6+/-4.0*	*-17.7+/-6.5*	*-11.8+/-0.9*

Supplemental Figure 2. Loss of *mir-60* promotes adaptive response against oxidative stress independently from known aging genes. Tables show numerical values and statistics for the survival curves shown in Fig 4; (**A**) for *daf-16*, (**B**) for *daf-2* and (**C**) for *skn-1*. 'EV' denotes Empty Vector, L4440 plasmid DNA used as a control in feeding RNAi. To further confirm the results of RNAi experiments, loss-of-function mutants of each gene were used. For *skn-1*, a mutation (*zu135* or *zu67* allele) causes lifespan reduction in *mir-60* mutants under the normal PQ 0 mM condition, which was comparable in that in the wild-type background. However, unexpectedly, both *skn-1* alleles rather significantly increased lifespans compared to the wild-type control under the PQ 5 mM conditions. Although the cause for this is currently under investigation (e.g. *skn-1* might have an unidentified role in adaptive response, such as enhancing hormesis effect), combining the results of our expression study that the levels of SKN-1 targets, such as *gst-4* and *gcs-1*[S1, S2], are not significantly changed in the *mir-60* loss background, we conclude that *skn-1* is dispensable for the *mir-60* loss to extend lifespan.

A

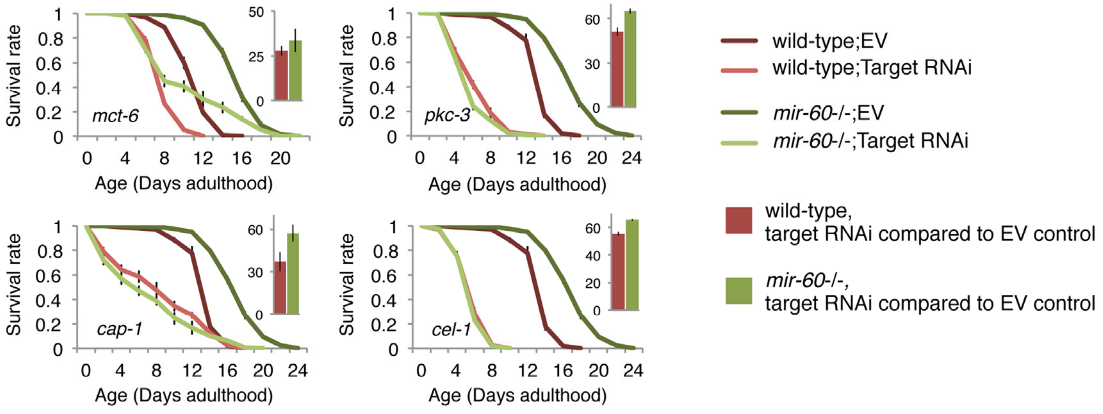

B

RNAi	Control (empty vector)		*apa-2*		*attf-3*		*W09D10.1*	
Strain	wild-type	*mir-60-/-*	wild-type	*mir-60-/-*	wild-type	*mir-60-/-*	wild-type	*mir-60-/-*
Number of replicate	3	3	3	3	3	3	3	3
Total number of dead worms	292	263	318	276	294	251	299	218
Mean adult lifespan+/-SEM	11.3+/-0.3	15.9+/-0.4	8.9+/-0.4	11.0+/-0.1	9.3+/-0.3	7.1+/-0.2	8.5+/-0.1	9.2+/-0.8
Rate of reduction+/-SEM (%)	0.0+/-2.8	0.0+/-2.6	21.6+/-3.1	30.5+/-0.4	17.8+/-2.3	55.0+/-1.2	24.5+/-1.2	41.9+/-4.9

RNAi	*ced-3*		*par-6*		*mct-6*	
Strain	wild-type	*mir-60-/-*	wild-type	*mir-60-/-*	wild-type	*mir-60-/-*
Number of replicate	3	3	3	3	3	3
Total number of dead worms	258	229	320	316	302	261
Mean adult lifespan+/-SEM	9.4+/-0.1	12.3+/-0.4	11.1+/-0.5	9.9+/-0.2	8.1+/-0.3	10.5+/-1.0
Rate of reduction+/-SEM (%)	16.7+/-0.6	22.6+/-2.5	2.2+/-4.1	37.9+/-1.1	27.9+/-2.5	33.6+/-6.4

RNAi	Control (empty vector)		*pkc-3*		*cel-1*		*cap-1*	
Strain	wild-type	*mir-60-/-*	wild-type	*mir-60-/-*	wild-type	*mir-60-/-*	wild-type	*mir-60-/-*
Number of replicate	3	3	3	3	3	3	3	3
Total number of dead worms	209	282	176	267	165	278	145	182
Mean adult lifespan+/-SEM	13.6+/-0.3	17.3+/-0.5	6.6+/-0.4	6.0+/-0.3	6.1+/-0.2	5.9+/-0.1	8.5+/-0.9	7.4+/-1.0
Rate of reduction+/-SEM (%)	0.0+/-2.1	0.0+/-2.6	51.2+/-3.0	65.2+/-1.8	55.3+/-1.5	65.6+/-1.5	37.2+/-6.8	56.9+/-5.8

C

```
pkc-3 3′ UTR
3′ ACU---UGAUCUUUUACACGUAUUAu 5′ miR-60-3p
    |||    ||||       ||  |||||||
5′ UGACACACUAACUCGUG-GCAUAAUC 3′ cel
5′ CC--CCAUUCGAGUGUG-GCAUAAUC 3′ cbr
5′ UGA--CACUAACUCGUG-GCAUAAUC 3′ crm
5′ UGA--UUAUGACACGUG-GCAUAAUU 3′ cjp

cap-1 3′ UTR
3′ ACUUGAUCUUUUACAC---------GUAUUAu  5′ miR-60-3p
        |||||     |||           |||||||
5′      CUAGA---CGUGCUUUACAACAUAAUAC 3′ cel
5′      UUAGA---CGUGCUUUACAACAUAAUAC 3′ cbr
5′      UUAGA---CGUGCUUUACAACAUAAUAC 3′ crm
5′      CUAGA---CGUGCUUUACAACAUAAUAU 3′ cbn

zip-10 3′ UTR Position 1
3′ ACUUGAUCUUUU---ACACGUAUUAu 5′ miR-60-3p
     |||              ||   ||||||
5′ CGAA C--CU--CUUUGC-CAUAAUU 3′ cel
5′ CUGCUCAAAAAAUCCUGC-CAUAAUU 3′ cbr
5′ CUACUCAAUU--UACUGC-CAUAAUU 3′ crm
5′ AUACUCACCU--CUGUGC-CAUAAUU 3′ cbn
5′  CCCCUAAU------UGCCCAUAAUU 3′ cjp

zip-10 3′ UTR Position 2
3′ ACUUGAUCUUUU--AC-AC-GUAUUAu 5′ miR-60-3p
       |||            ||  ||  ||||||
5′    CGCUCAUUCUU-UGCUGACAUAAUU 3′ cel
5′    UGCUCACUCUUGUGCUGACAUAAUU 3′ cbr
5′    UGCUCAUUUUU-UGCUGACAUAAUU 3′ crm
5′    AGCUCAAGUUU-UCAUGACAUAAUU 3′ cbn
5′    -----AAUCUC-CUCCGCCAUAAUU 3′ cjp

zip-10 the 2nd Exon
3′ ACUUGAUCUUUUACAC---------GUAUUAu 5′ miR-60-3p
        ||||||||||           ||||||
5′  AAGGUGGAGAAUGUGAAAAGUCUUCAUGAUG 3′ cel
```

Supplemental Figure 3. Longer lifespans induced by loss of *mir-60* are significantly shortened by RNAi inactivations against its predicted targets. (**A**) Additional results that are not shown in Fig 5 are presented. Small bar graphs indicate the percentage of lifespan reduction. Error bars represent SE calculated from 3-4 replicates. (**B**) A Table shows numerical values and statistics for the survival curves shown in Fig 5 and above. (**C**) miR-60-3p strand and its possible binding sites in 3' UTR of target candidates are shown as vertical lines. Conserved regions among *C. elegans* (*cel*)-related species, including *C. briggsae* (*cbr*), *C. remanei* (*crm*), *C. brenneri* (*cbn*) and *C. japonia* (*cjp*), are highlighted by while-colored letters on gray backgrounds.

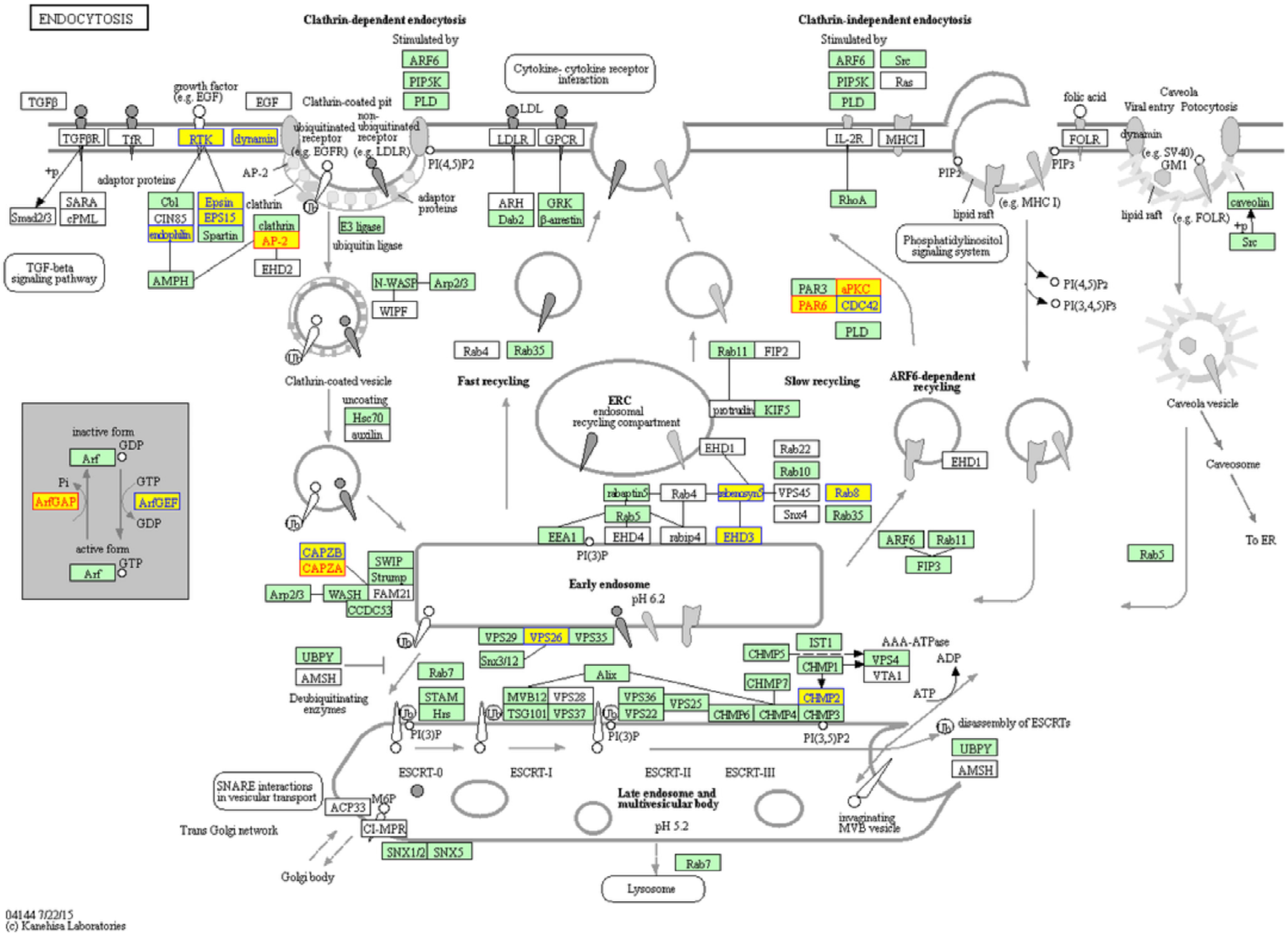

Supplemental Figure 4. Target genes of miR-60 seem to be involved in the endocytosis machinery. Genes predicted as miR-60 targets that function in the endocytosis machinery are highlighted by yellow on a KEGG map[S3]. Of those, 5 candidates confirmed by the genetic studies are shown in red, and the remaining computationally predicted candidates are shown in blue. Additional miR-60 target candidates, including, attf-3, mtm-6, mca-3 and tbc-2, are known to be involved in the endocytotic process, although these are not shown on this map.

A

Summary of sequencing reads:

Sample ID	Sample description	Total number of reads	Number of mapped reads	Percentage of mapped reads
s60A	*mir-60;spe-9*, Day 0, Replicate-1	5964,8540	5004,9257	83.9
s60B	*mir-60;spe-9*, Day 0, Replicate-2	6439,5856	5386,5193	83.6
speA	*spe-9*, Day 0, Replicate-1	6981,0077	5848,9365	83.8
speB	*spe-9*, Day 0, Replicate-2	6788,3067	5603,1268	82.5
speD7	*spe-9*, Day 7, PQ 5 mM	6787,1208	5626,2217	82.9
s60D7	*mir-60;spe-9*, Day 7, PQ 5 mM	7544,0022	6336,8602	84
s60D10	*mir-60;spe-9*, Day 10, PQ 5 mM	6699,6646	5479,5483	81.8

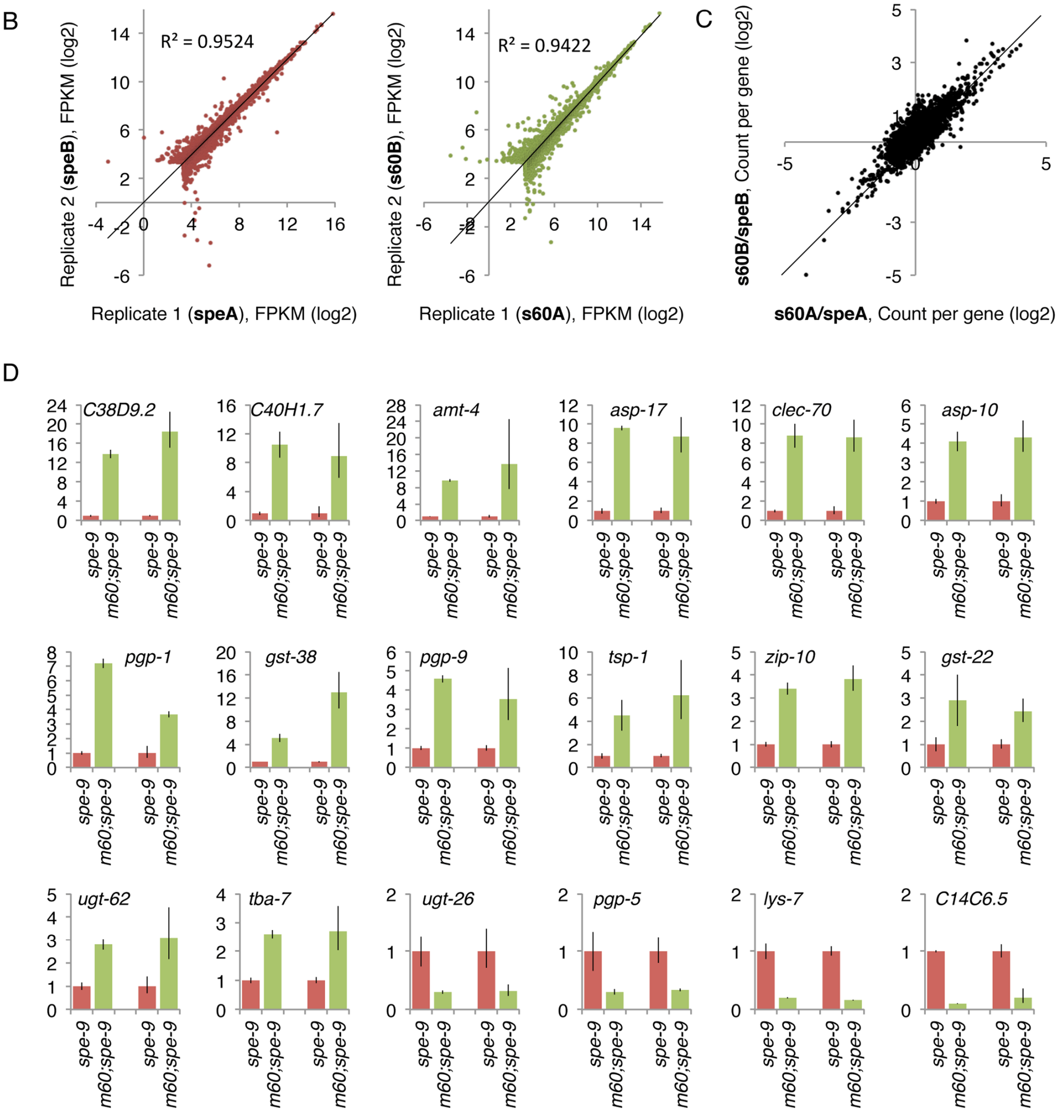

Supplemental Figure 5. RNA sequencing results were confirmed by biological replicates and qRT-PCR. (**A**) A Table shows the summary of RNA sequencing reads. (**B**) Gene expression was compared between each replicate of spe-9 and mir-60;spe-9 Day 0 control samples. Dots represent each transcript. The slopes for spe-9 (speA vs speB) and mir-60;spe-9 (s60A vs s60B) were 0.95244 and 0.9422, respectively, indicating that two biological replicates are very consistent. Transcripts with very low sequencing reads (less then 10 reads in FPKM in either 2 samples compared) were omitted. (**C**) The ratio in gene expression change (mir-60;spe-9 compared to the spe-9 control) was compared between the biological replicates based on counts per gene. Dots in upper right and in lower left on the panel mean that they have the same trend in expression change. Many dots scattered around the center mean that they are not significantly changed between spe-9 and mir-60;spe-9. These results demonstrate that most of the genes with significant expression changes have the same trend in two biological replicates. (**D**) The RNA sequencing results were further confirmed by qRT-PCR. Total RNA isolated from 3 independent trials of sample preparation, including the two replicates used for the RNA sequencing and one additional replicate separately prepared, were used for the qRT-PCR experiments in order to verify the results obtained by the RNA sequencing technology and also to confirm variation among biological samples. Error bars represent SE. Left and right two bars represent the results of RNA sequencing and qRT-PCR, respectively.

A

RNAi	Control (empty vector)		*ugt-62*		*spp-18*		*ugt-44*	
Strain	wild-type	*mir-60-/-*	wild-type	*mir-60-/-*	wild-type	*mir-60-/-*	wild-type	*mir-60-/-*
Number of replicate	3	3	3	3	3	3	3	3
Total number of dead worms	235	303	214	314	258	335	265	248
Mean adult lifespan+/-SEM	10.9+/-0.2	16.7+/-0.4	10.6+/-0.1	14.2+/-0.1	10.3+/-0.2	14.9+/-1	10.6+/-0.2	15+/-0.1
Rate of reduction+/-SEM (%)	0+/-1.9	0+/-2.5	2.6+/-0.5	15+/-0.7	6+/-1.9	10.8+/-6.2	3.1+/-2.2	10.7+/-0.6

RNAi	*oac-57*		*gst-22*		*pgp-9*		*amt-4*	
Strain	wild-type	*mir-60-/-*	wild-type	*mir-60-/-*	wild-type	*mir-60-/-*	wild-type	*mir-60-/-*
Number of replicate	3	3	3	3	3	3	3	3
Total number of dead worms	261	306	228	348	217	326	260	289
Mean adult lifespan+/-SEM	10.3+/-0.3	15.6+/-0.3	10.7+/-0.3	15.1+/-0.2	10.6+/-0.1	14.9+/-0.4	10.9+/-0.5	17.3+/-0.7
Rate of reduction+/-SEM (%)	5.8+/-3.1	6.6+/-1.6	2+/-2.8	9.8+/-1.2	3.4+/-1	11.3+/-2.4	-0.1+/-4.4	-3.6+/-4

RNAi	*asp-17*		*zip-10*	
Strain	wild-type	*mir-60-/-*	wild-type	*mir-60-/-*
Number of replicate	3	3	3	3
Total number of dead worms	257	269	235	392
Mean adult lifespan+/-SEM	11.1+/-0.3	16.4+/-0.7	9.9+/-0.2	13.2+/-0.3
Rate of reduction+/-SEM (%)	-1.9+/-2.3	2.3+/-4	9.6+/-2.3	21.2+/-1.8

B

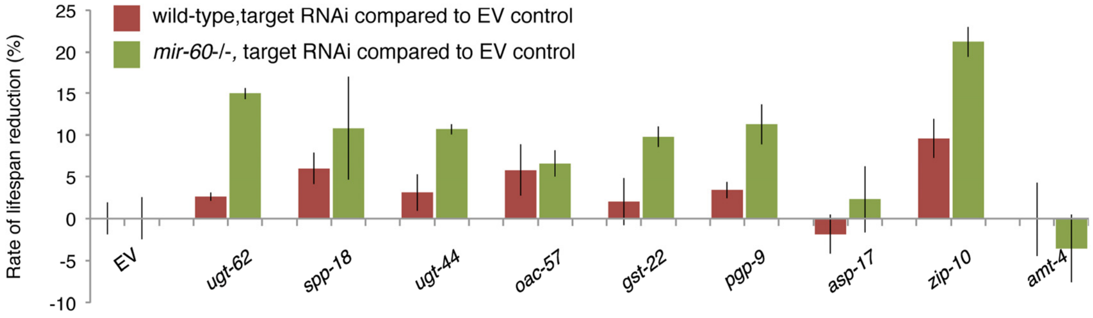

Supplemental Figure 6. Inhibiting *mir-60* loss-induced genes disrupts the adaptive response against oxidative stress. (**A**) A table shows numerical values and statistics for lifespan assays under the PQ 5 mM condition, where the effect of RNAi inactivation against genes up-regulated by the *mir-60* loss was examined. Survival curves for *zip-10* was plotted on a graph and is shown in Fig 7B. (**B**) In addition to *zip-10* RNAi, we have observed that additional RNAi inactivations, such as those against *ugt-62* and *pgp-9*, also significantly shorten the longer lifespan of *mir-60* mutants. However, unlike *zip-10*, RNAi against these genes was not highly reproducible; in 2 of 4 independent trials, the RNAi inactivations did not significantly abolish the *mir-60* loss-induced lifespan extension and resulted in comparable lifespan reduction compared to wild-type control animals exposed to these RNAi. Since many of the genes induced by the *mir-60* loss are members of families, such as *pgp-1*, *pgp-3* and *pgp-5*, which are all up-regulated by the *mir-60* loss, inactivation of each single gene may be less effective and stochastically generate variability in the lifespan phenotype because of genetic redundancy.

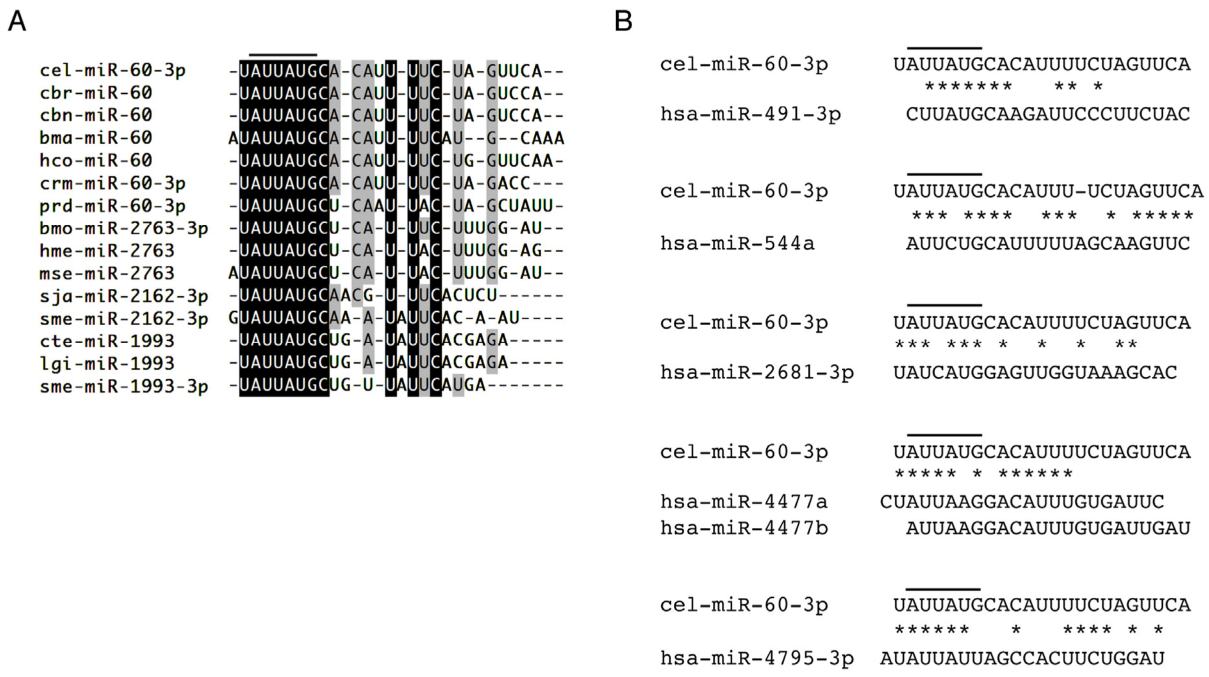

Supplemental Figure 7. *C. elegans* miR-60 (miR-60-3p) and its variants are conserved across species. (**A**) Each sequence shows mature miRNA strands in the 5' to 3' directions from left to right. 6 nucleotides (AUUAUG; position 2-7) highlighted by an underline represent the seed region. *C. elegans* miR-60 (cel-miR-60-3p) is highly conserved in nematode species and some insect species. cel: *Caenorhabditis elegans* (nematode), cbr: *Caenorhabditis briggsae* (nematode), cbn: *Caenorhabditis brenneri* (nematode), bma: *Brugia malayi* (nematode), hco: *Haemonchus contortus* (nematode), crm: *Caenorhabditis remanei* (nematode), prd: *Panagrellus redivivus* (nematode), bmo: *Bombyx mori* (Insect; silk worm), hme: *Heliconius melpomene* (Insect; butterfly), mse: *Manduca sexta* (Insect; hornworm), sja: *Schistosoma japonicum* (Roundworm), sme: *Schmidtea mediterranea* (Planarian), cte: *Capitella teleta* (Polychaete worm), lgi: *Lottia gigantea* (Sea snail). (**B**) Human mature miRNAs having *C. elegans* miR-60-like seed regions are shown.

SUPPLEMENTAL TABLES

Please browse the Full Text version to see the Supplemental Tables of this manuscript:

Supplemental Table 1. A list of computationally predicted targets of miR-60.
Supplemental Table 2. Processed RNA sequencing results represented as FPKM.
Supplemental Table 3. A list of genes differentially expressed between the control and *mir-60* loss background at Day 0 adulthood examined by the DEseq program.
Supplemental Table 4. Detailed results of GSEA analysis for genes affected by the mir-60 loss.
Supplemental Table 5. Summary of RNA sequencing – the number of gene counts in each sample.
Supplemental Table 6. *C. elegans* strains used in this study.

SUPPLEMENTAL METHODS

Suppressor screens to identify the targets of miR-60

We sought RNAi inactivations against target candidates that suppressed the *mir-60* loss-induced lifespan extension under the mild and long-term oxidative stress. Multiple algorithms, including TargetScan [S4], miRanda [S5], PicTar [S6], mirWIP [S7], RNA22[S8] and DIANA microT [S9], were combined to generate a list of possible miR-60 targets (Supplemental Table 1). Of those, candidates predicted by multiple programs, which consist of approximately 400 genes, were examined for their effect on lifespan of *mir-60* mutants. Developmentally synchronized *mir-60* mutants were cultured on each RNAi bacteria, including empty vector control, from L1 stage at 20 °C in 24-well plates. PQ (5 mM in the final concentration) and FUDR were supplemented when they reached the young adult stage. Animals were checked for survival at Day 10-12, where most of wild-type animals treated with the control RNAi were essentially dead, while many of the *mir-60* mutants treated with the control RNAi were still survived. We checked survival of *mir-60* mutants, which were exposed to each candidate RNAi, and briefly scored their death rate, 50%, 75% and 100%. The experiments were repeated 3 times independently, including 2 replicates in each trial. RNAi inactivations causing significant death (>50%) in 3 or more in the total 6 replicates were considered for further analyses. These screens generated approximately 50 potentially positive candidates. We then excluded those causing developmental arrest or obvious sickness in early adulthood. For the remaining candidates, we performed conventional lifespan assays under the PQ 5 mM condition multiple times, eventually identifying 9 strong candidates, including the endocytosis-related genes (Fig 5A and Supplemental Fig 3A/B).

Transcriptome analysis of mir-60 mutants

To identify genes that respond to the *mir-60* loss, RNA expression profiles were examined between the *mir-60* mutant and its control animals using high-throughput sequencing. In this study, we used *spe-9*(*hc88*), a temperature-sensitive sterile strain, which has been shown in previous studies to have a lifespan similar to wild-type and used in gene expression studies to reduce the effect of RNA contamination from young progeny (see main text for references). We initially tried to use *glp-4*(*bn2*) mutants, which are also known to have a temperature-sensitive sterility with a wild-type lifespan [S10]; however, since we found that 20-30% of *glp-4* animals were dead by vulval-bursting around Day 6-7, the mid-age at 23.5 °C, we thought that *glp-4* animals are not suitable for a longitudinal experiments, such as gene expression study during aging, and we have decided to use *spe-9*(*hc88*) mutant instead. We initially performed lifespan assays of *spe-9* single and *mir-60;spe-9* double mutant animals under the PQ 5 mM condition at 23.5 °C to determine 50% survival time points in each strain; 7.5+/-0.3 for *spe-9* and 10.1+/-0.3 for *mir-60;spe-9*. Total RNA was purified from both *spe-9* and *mir-60;spe-9* strains at the Day 0 young adult stage (just before PQ exposure) twice independently for biological replicates. In addition, total RNA was also purified at 50% survival time points – Day 7 for both strains and Day 10 for only *mir-60;spe-9*. cDNA libraries were made for these 7 samples (see below for a sample list), and each was indexed using the Illumina's library preparation kit and sequenced on 2 lanes of flow cells on the HiSeq 2000 platform. Sequencing reads were processed as described in Experimental Procedures. In this study we essentially focused on those at the Day 0 stage between *spe-9* and *mir-60;spe-9* strains since differences in expression between these strains at later stages were less significant. Expression levels of genes in all samples examined are shown in Supplemental Table 5 as a reference. Raw sequence data and processed reads represented as FPKM (Supplemental Table 2) are deposited to a public database GEO with the accession number GSE83239.

Sample IDs	Description
speA	*spe-9*, Day0, Replicate-1
speB	*spe-9*, Day0, Replicate-2,
s60A	*mir-60*;*spe-9*, Day0, Replicate-1
s60B	*mir-60*;*spe-9*, Day0, Replicate-2
speD7	*spe-9*, Day7
s60D7	*mir-60*;*spe-9*, Day7
s60D10	*mir-60*;*spe-9*, Day10

SUPPLEMENTAL REFERENCES

S1. Kahn NW, Rea SL, Moyle S, Kell A, and Johnson TE. Proteasomal dysfunction activates the transcription factor SKN-1 and produces a selective oxidative-stress response in Caenorhabditis elegans. The Biochemical journal 2008. 409:205-13.

S2. Tullet JM, Hertweck M, An JH, Baker J, Hwang JY, Liu S, Oliveira RP, Baumeister R and Blackwell TK. Direct inhibition of the longevity-promoting factor SKN-1 by insulin-like signaling in C. elegans. Cell. 2008; 132:1025-38.

S3. Kanehisa M, Sato Y, Kawashima M, Furumichi M, and Tanabe M. KEGG as a reference resource for gene and protein annotation. Nucleic acids research. 2016. 44:D457-62.

S4. Lewis BP, Burge CB, and Bartel DP. Conserved seed pairing, often flanked by adenosines, indicates that thousands of human genes are microRNA targets. Cell. 2005; 120:15-20.

S5. Betel D, Wilson M, Gabow A, Marks DS, and Sander C. The microRNA.org resource: targets and expression. Nucleic acids research. 2008; 36:D149-53.

S6. Lall S, Grun D, Krek A, Chen K, Wang YL, Dewey CN, Sood P, Colombo T, Bray N, Macmenamin P, et al. (2006). A genome-wide map of conserved microRNA targets in C. elegans. Current biology . 2006; 16:460-71.

S7. Hammell M, Long D, Zhang L, Lee A, Carmack CS, Han M, Ding Y, and Ambros V. mirWIP: microRNA target prediction based on microRNA-containing ribonucleoprotein-enriched transcripts. Nature methods. 2008; 5:813-19.

S8. Miranda KC, Huynh T, Tay Y, Ang YS, Tam WL, Thomson AM, Lim B, and Rigoutsos I. A pattern-based method for the identification of MicroRNA binding sites and their corresponding heteroduplexes. Cell. 2006; 126:1203-17.

S9. Paraskevopoulou MD, Georgakilas G, Kostoulas N, Vlachos IS, Vergoulis T, Reczko M, Filippidis, C, Dalamagas T, and Hatzigeorgiou AG. DIANA-microT web server v5.0: service integration into miRNA functional analysis workflows. Nucleic acids research. 2013; 41:W169-73.

S10. Curran SP, Wu X, Riedel CG, and Ruvkun G. A soma-to-germline transformation in long-lived Caenorhabditis elegans mutants. Nature. 2009; 459:1079-84.
